# Antitumor Therapy Targeting the Tumor Microenvironment

**DOI:** 10.1155/2023/6886135

**Published:** 2023-03-03

**Authors:** Yuewen Gao, Zhengwu Pan, Hongqi Li, Fei Wang

**Affiliations:** ^1^Department of Gynecology, Shandong Provincial Hospital, Cheeloo College of Medicine, Shandong University, Jinan, Shandong 250021, China; ^2^Department of Gynecology, Shandong Provincial Hospital Affiliated to Shandong First Medical University, Jinan, Shandong 250021, China

## Abstract

The development and progression of tumors in human tissues extensively rely on its surrounding environment, that is, tumor microenvironment which includes a variety of cells, molecules, and blood vessels. These components are modified, organized, and integrated to support and facilitate the growth, invasion, and metabolism of tumor cells, suggesting them as potential therapeutic targets in anticancer treatment. An increasing number of pharmacological agents have been developed and clinically applied to target the oncogenic components in the tumor microenvironment, and in this review, we will summarize these pharmacological agents that directly or indirectly target the cellular or molecular components in the tumor microenvironment. However, difficulties and challenges still exist in this field, which will also be reported in this literature.

## 1. Introduction

**Figure 1:**
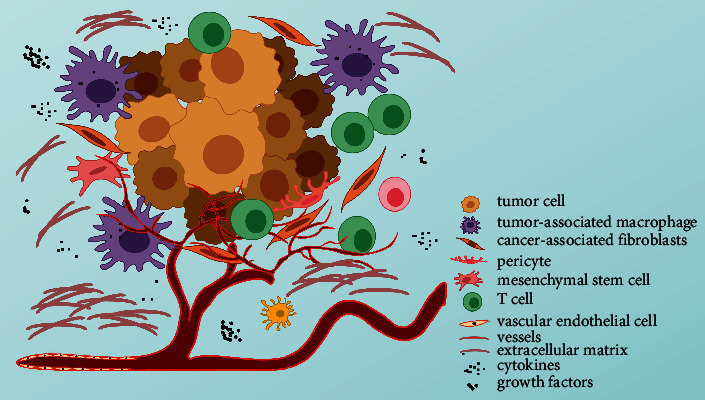
Schematic representation of the components in the TME: TME is mainly composed of tumor cells, their surrounding immune cells and inflammatory cells, cancer-associated fibroblasts, and nearby interstitial tissues, microvessels, as well as various cytokines and chemokines, which is a complex comprehensive system.

The development and progression of cancer enormously depend on the TME, which typically contains numerous cell types, including fibroblasts, endothelial cells, pericytes, and diverse immune cells. Together with tumor cells, these cells are embedded in the extracellular matrix (ECM) such as cytokines and growth factors [1]. These cells and ECM components dynamically interact with the tumor cells, regulating tumor growth, progression, invasion, and metastasis (Figure 1). In recent decades, with the in-depth study of TME, the mystery of the interplay between TME and tumor cells has been gradually unraveled and therapeutically targeting TME has emerged as a promising anticancer treatment strategy. Herein, we briefly summarize the essential cellular and molecular components of TME with an emphasis on pharmacological methods against these cells and ECM as anticancer treatments. Some current challenges and concerns associated with TME-targeted therapies will be discussed as well.

## 2. Strategies Targeting TME

### 2.1. Targeting Tumor Angiogenesis Mainly through the VEGF-VEGFR Signaling Pathway

The tumor-associated neovasculature, generated through the process termed angiogenesis, satisfies the acquisition of nutrients and oxygen as well as the evacuation of wastes and carbon dioxide for the tumor cells. Angiogenesis is mainly regulated by the proangiogenic factors and antiangiogenic factors. When the two types of regulators are balanced, the “angiogenic switch” is in the “off” state. However, when the proangiogenic factors become dominant, angiogenesis can be triggered [2]. Hypoxia is one of the major angiogenetic stimuli, which activates angiogenesis through the production of hypoxia-inducible factor 1 (HIF-1) [3]. Under the stimulation of hypoxia, HIF-1 generated by tumor cells facilitates the secretion of various proangiogenic factors, such as fibroblast growth factor (FGF), platelet derived growth factor (PDGF), vascular endothelial growth factor (VEGF), and angiopoietin-1 (Ang-1), thus promoting the proliferation, migration, and transformation of vascular endothelial cells [4]. The constituents of the ECM including elastin, collagen, laminin, fibronectin, and proteoglycans, are the macromolecules secreted by tumor cells and tumor-associated fibroblasts, which can not only support and protect tumor cells but also promote tumor invasion and metastasis [5, 6].

Among these proangiogenic VEGF factors, VEGF-A is the most extensively studied and well-known target of antiangiogenesis treatment [7]. VEGF-A binds to its receptor VEGF receptor 1 (VEGFR1) and VEGF receptor 2 (VEGFR2, the major signaling receptor for angiogenesis) that are predominantly expressed on vascular endothelial cells, thus activating VEGF-VEGFR signaling [8]. VEGF-VEGFR signaling activation promotes the proliferation of endothelial cells, contributing to the formation of new blood vessels characterized by increased permeability within the tumors [8, 9]. Therefore, VEGF-VEGFR signaling has emerged as an appealing anticancer therapeutic target.

**Figure 2:**
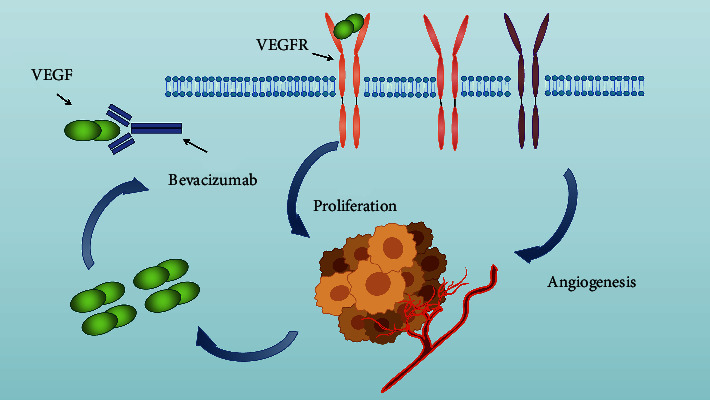
Mechanism of bevacizumab against tumor: bevacizumab inhibits cancer cell proliferation and tumor neovascularization by blocking VEGF/VEGFR.

Bevacizumab, a humanized monoclonal antibody against VEGF-A, can bind to VEGF-A and inhibit its activity through suppressing receptor binding, endothelial cell proliferation, and neovasculature formation, thus decelerating tumor growth (Table 1) (Figure 2) [34–36]. Furthermore, bevacizumab can improve the vascular structure within tumors and normalize abnormal blood vessels by inhibiting the activity of VEGF-A, leading to increased blood vessel permeability, improved local hypoxia condition, and enhanced anticancer agent delivery [37, 38]. The study of Soria et al. has identified bevacizumab in combination with standard platinum-based chemotherapy which significantly prolonged overall survival and progression-free survival (PFS) in patients with nonsmall cell lung carcinoma [10]. In addition, bevacizumab also improves the outcome of patients diagnosed with renal cancer [11], metastatic colorectal cancer [12–15], and metastatic breast cancer [16]. However, further clinical trials should focus more on improving the efficacy of bevacizumab, including exploring the optimal population, optimal dose, and optimal timing for bevacizumab-based therapy.

Ramucirumab, a fully human monoclonal antibody, specifically binds to VEGFR2 with high affinity, thus blocking the binding of VEGFR2 ligands which include VEGF-A, VEGF-C, and VEGF-D and contributing to the inhibition of VEGFR2-mediated tumor angiogenesis (Table 1) [39, 40]. Therefore, ramucirumab can block the proliferation and migration of vascular endothelial cells and ultimately suppress the angiogenesis [41]. Ramucirumab has been approved for the treatment of diverse malignancies, including gastric cancer, nonsmall cell lung carcinoma, and metastatic colorectal cancer [17, 18].

The VEGF inhibitor, aflibercept, is a recombinant fusion protein that is composed of the ligand-binding element from the extracellular domain of VEGFR1 and VEGFR2 and the Fc segment of human immunoglobulin G1 (IgG1) (Table 1) [42, 43]. Through binding to VEGFs, aflibercept functions as a “VEGF trap” and inhibits the neovasculature formation induced by VEGFs and thereby “starving” tumors [42, 44]. Aflibercept in combination with fluorouracil, leucovorin, and irinotecan (FOLFIRI) significantly improved overall survival and PFS in metastatic colorectal cancer patients who were previously treated with oxaliplatin [45]. In 2012, aflibercept, in combination with the FOLFIRI regimen, was approved by the United States Food and Drug Administration (FDA) for the treatment of patients with metastatic colorectal cancer.

Tyrosine kinase inhibitors, pazopanib, sunitinib, sorafenib, and regorafenib, are multitarget kinase inhibitors that can potently bind and diminish the activities of VEGFRs, thereby inhibiting tumor angiogenesis and growth [46–52]. Pazopanib is approved by FDA as an anticancer medication for metastatic renal cell carcinoma (RCC) and advanced soft tissue sarcomas (Table 1) [21, 22]. Sunitinib and sorafenib are also approved for the treatment of RCC (Table 1). Additionally, sunitinib can also be used in patients with advanced gastrointestinal stromal tumors (GIST) after disease progression or intolerance to imatinib [23, 24], and sorafenib is also approved for the treatment of patients with inoperable hepatocellular carcinoma [25, 26]. Regorafenib is approved to treat patients with metastatic colorectal cancer that progresses after previous antitumor therapy [27], as well as patients with advanced GIST after the failure of other anticancer therapy [28] and patients with hepatocellular carcinoma who progress on sorafenib treatment (Table 1) [53].

Although these kinase inhibitors exert powerful anticancer effects on multiple malignancies, the development of resistance against these agents tremendously limits the benefit that patients can achieve from the therapy. The rapamycin analog, everolimus, has been approved by the FDA as a treatment of RCC refractory to sunitinib or sorafenib [54]. Everolimus inhibits tumor growth not only through affecting the PI3K/Akt/mTOR pathway but also through blocking tumor angiogenesis via downregulating the expression of HIF-1 and VEGFs (Table 1) [29, 55].

### 2.2. Targeting Hypoxia

Hypoxia impacts the tumor growth, progression, and angiogenesis mainly through the transcriptional factor HIF-1*α*. Topotecan (TPT) is a topoisomerase I inhibitor that has been approved for the treatment of small cell lung cancer, ovarian cancer, and cervical cancer. TPT can interfere with the process of DNA replication in tumor cells via slowing down the relegation activity of topoisomerase I and promoting the conversion of topoisomerase I cleavage complexes into DNA damage by replication-fork collision and transcription (Table 1) [56]. Consequently, this DNA damage can trigger tumor cell apoptosis [56, 57]. Strikingly, TPT can also inhibit HIF-1*α* transcriptional activity and HIF-1*α* protein accumulation by affecting its translation [58, 59]. TPT can activate the deacetylase activity of sirtuin 1 (SIRT1) and lead to the degradation of HIF-1*α* through deacetylation. Therefore, TPT can influence the angiogenesis of tumors and the metabolism of tumor cells, thus blocking the tumor progression [60].

### 2.3. Target ECM through Destruction and Remodeling

Collagen is the main structural element of the matrix, of which type I collagen is the main component of tumor desmoplasia and is relative to the survival and metastasis of many types of tumor cells [61]. In normal tissues, the basement membrane is rich in collagen and laminin, which separates the endothelial and epithelial layers from the stroma. In tumors, the basement membrane becomes thinner due to the reduction of type IV collagen, which is conducive to tumor invasion and metastasis [62]. Proteoglycan (PG) is the main component of the extracellular matrix, including many types such as extracellular proteoglycans, pericellular-basement proteoglycans, cell surface proteoglycans, intracellular proteoglycans, and so on, which interacts with various growth factors, cytokines, chemokines, etc., to regulate and control the proliferation and migration of tumors [63]. As the progression of tumors, the extracellular matrix is remodeled and the structure of the collagen scaffold in the tumor changes seriously, which is conducive to the angiopoiesis and the migration of the tumor cells [64].

Activated fibroblasts located within the stroma of tumors are called tumor-associated fibroblasts (CAFs). CAFs are mainly derived from resident fibroblasts that can be activated by PDGF, FGF, and transforming growth factor *β* (TGF-*β*) released by tumor cells [65, 66]. Secondly, epithelial or endothelial cells in tumors can be transformed into CAFs through epithelial-mesenchymal transitions (EMT) and endothelial-mesenchymal transitions (EndMT) [67, 68]. In addition, studies have confirmed that recruited mesenchymal stem cells (MSCs) derived from bone marrow are also the origin of CAFs [69, 70]. Compared with normal fibroblasts, CD34 expression is absent in CAFs, and smooth muscle actin *α* (*α*-SMA) is expressed. Moreover, the molecular markers of CAFs include platelet-derived growth factor receptor (PDGFR), vimentin, fibroblast activation protein (FAP), and CAF specific protein (FSP1/S100A4) [71, 72].

CAFs can express fibroblast activating protein *α* (FAP), while normal fibroblasts do not. Therefore, it is speculated that drugs targeting FAP can inhibit the growth and metastasis of tumors through inhibiting CAFs. However, several clinical trials targeting FAP with human-derived monoclonal antibodies have failed to produce clinical benefits in colon cancer and non-small cell lung cancer [73].

ECM components exert a supportive and protective effect on tumor cells, and many processes of signal transduction also occur in ECM. ECM components have a profound influence on the tumor growth, progression, invasion, and metastasis, indicating them as attractive anticancer therapeutic targets. Currently, antitumor therapy targeting ECM mainly consists of two aspects: the destruction of ECM and remodeling of ECM.

#### 2.3.1. Destruction of ECM

*(1) Angiotensin receptor blockers*. Angiotensin receptor blockers (ARBs) such as losartan, valsartan, and their analogs are capable of reducing blood pressure through blocking the angiotensin II type I receptors (AGTR1) (Table 2) [79]. Additionally, numerous studies have demonstrated that ARBs can inhibit the tumor proliferation, promote the tumor cell apoptosis, and impede tumor metastasis as well as angiogenesis [74, 80, 81]. Through inhibiting AGTR1, losartan and its analogs can decrease the levels of decrease transforming growth factor-*β* (TGF-*β*) activators such as thromboplastin-1 (TSP-1) to reduce the quantity of TGF-*β*, thus inhibiting the synthesis of type I collagen derived from cancer-associated fibroblasts (CAFs) to reduce the proliferation of connective tissues [82]. The delivery of chemotherapeutic drugs toward tumor cells can be enhanced by such an antifibrotic effect [82]. The study of Busby et al. identified that ARBs can effectively reduce the mortality of patients with gastroesophageal cancer [74]. The study of Nakai et al. demonstrated that patients with pancreatic ductal adenocarcinoma who were treated with ARBs had an overall survival time of approximately 6 months longer than those who were not treated with ARBs [75]. As well as the study of Coulson et al. confirmed that ARBs restrain the occurrence and development of breast cancer by inhibiting the AGTR1 [76]. Moreover, the study by Jain has shown that ARBs can normalize the blood vessels and collagen matrix in tumors by blocking TGF-*β* and improve the efficacy of liposomal doxorubicin [83].

*(2) Enzymes that degrade ECM*. A number of enzymes, for example, matrix metalloenzymes (MMPs), hyaluronidases, and collagenases, are capable of degrading the ECM as well as loosening the ECM structure, contributing to improved anticancer drug delivery. MMPs can degrade the entire components in ECM, including collagen and proteoglycans, which promote the delivery and convection of drugs [84]. However, MMPs can also promote the angiogenesis within the tumors by accelerating the release of VEGFs, which is conducive to the growth, progression, invasion, and metastasis of tumors. For this reason, the application of MMPs in the treatment of cancer is still under controversy [85]. The tetracycline analogue Col-3, as an inhibitor of MMPs, can inhibit the production and activation of MMPs, particularly MMP-2 and MMP-9, thereby preventing the degradation of ECM and impeding the progression and metastasis of tumors (Table 2) [86, 87].

Collagen in ECM can significantly block the delivery of macromolecular drugs to tumor cells in vivo [88]. The study of McKee et al. proved that, despite increasing the risk of tumor metastasis, collagenase can enhance the diffusion of macromolecular drugs into the tumor stroma by destroying the collagen structure in ECM, thus playing a significant role in promoting tumor therapy [89]. Hyaluronic acid (HA) that is responsible for disorders associated with high interstitial fluid pressure (IFP) is abundant in ECM. HA leads to vascular collapse and affects the delivery and diffusion of micromolecular drugs [90]. Through degrading HA in ECM, hyaluronidase is able to rapidly reduce IFP, thus facilitating chemotherapeutic drugs to reach the targets of tumor cells at higher concentration [91]. Therefore, the combination of hyaluronidase with cytotoxic drugs such as paclitaxel and gemcitabine immensely improves anticancer efficacy in patients [91]. Due to the common existence of collagen and HA all over the human body, the use of these enzymes as anticancer therapeutics is likely to cause systemic adverse reactions; therefore, their application remains challenging in clinical practice [92].

#### 2.3.2. Remodeling of ECM

Tumor ECM is extremely dense and difficult to penetrate which is attributed to the proliferation and expansion of connective tissues. This condition can be modified by remodeling the ECM and inducing the normalization of ECM. Metformin, a biguanide antihyperglycemic agent, is the first-line treatment for type 2 diabetes [93]. Of note, metformin has been shown to act as an anticancer agent by reprogramming hepatic stellate cells (PSCs) to reduce the production of components such as type I collagen and HA in ECM (Table 2) [94]. In addition, metformin can reduce the production of connective tissue cytokines, the recruitment of tumor-associated macrophages (TAMs) as well as the polarization of M2 macrophages. Therefore, metformin is conducive to preventing the invasion and metastasis of tumors as a consequence of the inhibition of ECM remodeling and epithelial-mesenchymal transitions (EMT) [94]. Metformin is also a mitochondrial respiratory poison that can activate adenosine monophosphate-activated protein kinase (AMPK), which improves hypoxia within the tumor by decreasing the oxygen consumption [95, 96]. However, multiple clinical trials indicated that the effect of metformin in the treatment of various cancers is limited [97, 98].

### 2.4. Targeting Immune Cells

The macrophages that infiltrate around the tumors are referred to as tumor-associated macrophages (TAMs), which exert immunosuppressive functions [99]. These macrophages are recruited to the tumor tissue by various chemokines released from fibroblasts and tumor cells, for example, CC chemokine ligand 2 (CCL2), CC chemokine ligand 5 (CCL5), and CXC chemokine ligand8 (CXCL8) [100–102]. TAMs play a vital role in promoting the tumor angiogenesis by releasing proangiogenic factors such as TGF-*β*, PDGF, and VEGF. Furthermore, TAMs produce proteases such as urokinase-type plasminogen, plasmin, and MMPs (for example, MMP-1, MMP-2, MMP-3, MMP-9, and MMP-12) that can promote tumor angiogenesis and can directly remodel ECM. Lymphatic endothelial growth factors and vascular endothelial growth factor receptor 3 (VEGFR3) generated by TAMs promote lymphangiogenesis, release the epidermal growth factor (EGF) which can interact with colony stimulating factor 1 (CSF-1) secreted by tumor cells, and degrade proteins in the ECM through proteases such as MMP-2 and MMP-9, all of which are beneficial to facilitate the invasion and metastasis of tumors [103, 104].

Arginase 1, TGF-*β*, and interleukin-10 (IL-10) derived from TAMs play a significant role in tumor immunosuppression. Arginase 1 mainly produces arginine metabolites including polyamine and proline, which results in the dysregulation of the signals of T-cell receptor (TCR), and ultimately induces CD8+ T cell inactivity [105]. TFG-*β* plays an immunosuppressive role in multiple ways, which include promoting the differentiation of CD4+ T cells into Th2 cells, promoting the activation of TAMs, reducing the migration of dendritic cells, inhibiting the effects of natural killer (NK) cells, and inhibiting the cytotoxicity of CD8+ T cells [106]. With respect to IL-10, on the one hand, it inhibits the expression of the potential antitumor cytokines such as interleukin-12 (IL-12); on the other hand, it impedes the maturation of dendritic cells (DCs) and promotes macrophages to differentiate, and then the antigen presentation will be inhibited. In addition, IL-10 also blocks the release of interferon-*γ* (INF-*γ*), thus promoting immune escape [107, 108]. TAMs can also release a variety of immunosuppressive factors, including indoleamine 2, 3-dioxygenase (IDO), IL-10, and prostaglandin E2 (PGE2), which are conducive to immunosuppression [109].

A heterogeneous population composed of immature myeloid cells and myeloid-cell progenitor cells is defined as myeloid-derived suppressor cells (MDSCs), including immature dendritic cells, immature granulocytes, and immature macrophages [110]. MDSCs are recruited into the surrounding environment of the tumor by chemokines (for example, CCL2, CCL5, CXCL1, CXCL5, CXCL6, CXCL8, and CXCL12), followed with the activation of MDSCs by the granulocyte-macrophage colony stimulating factor (GM-CSF), granulocyte-colony stimulating factor (G-CSF), and VEGF [111–113]. Arginine is one of the chemicals that is essential for T-cells to complete the cell cycle. MDSCs degrade arginine by secreting arginase-1, thus inhibiting the activity of CD8+ T-cells by preventing the completion of the cell cycle [114, 115]. Monocytic-MDSCs (M-MDSCs) can produce nitric oxide (NO) and reactive oxygen species (ROS) through inducible nitric oxide synthase (iNOS) and NADPH oxidase (NOX2), resulting in oxidative stress in the TME, thus affecting the activity of CD8+ T-cells. Polymorphonuclear MDSCs (PMN-MDSCs, also known as granulocytes) inhibit CD8+ T-cells mainly via releasing ROS [116]. In addition, MDSCs can promote the transformation of initial CD4+ T-cells into induced regulatory T-cells (iTreg cells) which can inhibit the function of NK cells by secreting IL-10 and TGF-*β*.

In 1995, a cluster of CD4+ T-cells highly expressing the IL-2 receptor *α*-chain (CD25) and under the regulation of forkhead box protein 3 (Foxp3) was identified. Moreover, these cells with high immunosuppressive activity are termed regulatory T-cells (Treg cells) [117, 118]. Treg cells are abundant in the tumor microenvironment, in which their high-density infiltration is generally associated with poor cancer prognosis [119]. Tregs cells are mainly divided into natural Tregs cells and induced Tregs cells according to their origin. Treg cells present in the TME are mainly induced Tregs cells, which are derived from peripheral naive CD4+ T-cell precursors under tolerogenic conditions and can upregulate the expression of Foxp3 [119, 120]. Treg cells express CC chemokine receptor 4 (CCR4), the receptors of CC chemokine ligand 22 (CCL22), and can migrate to CCL22 derived from tumor cells and tumor associated macrophages in the TME, thus realizing the recruitment of Treg cells [121]. In addition, studies have shown that hypoxia can induce the expression of CC-chemokine ligand 28 (CCL28), which binds to the receptor CC chemokine receptor 10 (CCR10) on Treg cells to promote the recruitment of Treg cells [122].

**Figure 3:**
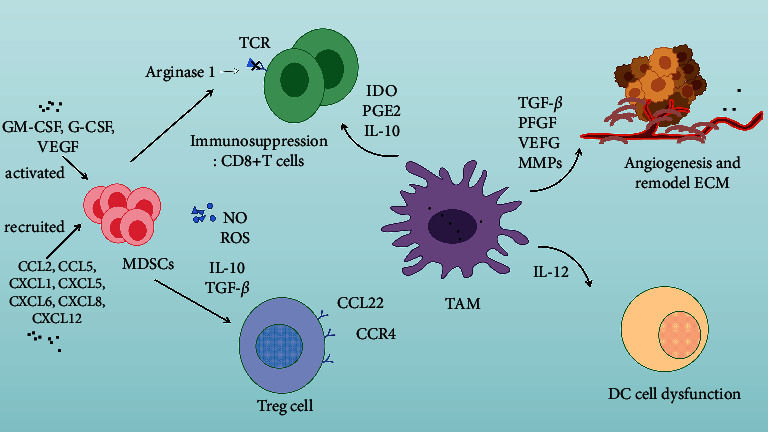
Tumor immune microenvironment: the lymphocytes infiltrating into the tumor mediated the immunosuppressive tumor microenvironment, helped the tumor cells achieve immune escape, and promoted the malignant development of the tumor, in which TAM and Treg cells played a major role.

Treg cells regulate the immune system through a number of mechanisms. For instance, Treg cells impede the effects of effector T-cells by secreting cytokines such as TGF-*β*, IL-10, and interleukin-35 (IL-35) [119]. Additionally, cytolysis is induced by granzyme B, the tumor necrosis factor-related apoptosis-inducing ligand (TRAIL) pathway, and galactosis-1, to induce apoptosis of target effector cells. Notably, Cao et al. demonstrated that granzyme B and perforin derived from Treg cells possess the ability of inhibiting NK cells and the cytotoxic effect of CD8+ cells to eliminate tumors [123]. Treg cells also induce DCs to up-regulate indolylamine 2, 3-dioxygenase (IDO) through expressing cytotoxic T lymphocyte antigen 4 (CTLA-4), thereby inhibiting the function of effector T-cells by affecting tryptophan metabolism [124, 125]. The mechanism by which immune cells play a role in the tumor microenvironment can be referred to in Figure 3.

In recent years, immune therapy has been developed as a powerful weapon against cancer. Anticancer immune therapy is mainly divided into therapy targeting the TAMs, adoptive cell therapy (ACT), and targeted therapy.

#### 2.4.1. Antitumor Therapy against TAMs

*(1) Inhibit the recruitment of TAMs*. The cytokine CCL2, which is identified as highly expressed in diverse tumors, induces mononuclear cells in the blood to migrate to the tumor tissue and transform into TAMs [126, 127]. Moreover, the elevated expression of CCL2 is closely associated with the polarization of M2 macrophages. Bindarit, a small anti-inflammatory molecule that blocks the recruitment of TAMs by inhibiting the expression of CCL2, can inhibit the progression of tumors and relieve pain in cancer patients (Table 3) [128, 146]. The study of Liu et al. suggested that bindarit may exert a potential antitumor effect by targeting I*κ*B*α* and p65 [128].

Macrophage colony stimulating factor 1 (CSF-1) recruits TAMs to tumors by binding to the macrophage colony stimulating factor 1 receptor (CSF1R). The inhibitors of CSF-1 or CSF1R can suppress the progression of tumors by inhibiting the recruitment of TAMs [147]. For example, CSF1R inhibitors such as GW2850 and PLX3397 are able to block CSF-1/CSF1R signaling and inhibit the recruitment of TAMs [148, 149]. Furthermore, GW2850 and PLX339 can kill tumor cells with high expression of CD206 directly or reprogram TAMs for antitumor therapy [150].

*(2) Reverse the TAMs phenotype*. It is widely believed that the subtype M1 macrophages have antitumor functions, while M2 macrophages have a protumor effect. Therefore, reversing or transforming M2 macrophages to M1 macrophages is considered as a method to inhibit the growth, progression, invasion, and metastasis of tumors. When TGF-*β* is inhibited, toll-like receptor 7 (TLR7) can reprogram TAMs to promote transformation into M1 macrophages, impeding the progression of tumors. Celecoxib, an inhibitor of cyctoxase II (COX-2), promotes the transformation of TAMs into M1 macrophages via inducing interferon-C (IFN-C) (Table 3) [151]. Another COX-2 inhibitor, etodolac, blocks the differentiation of monocytes into M2 macrophages, thereby inhibiting the growth and metastasis of tumors (Table 3) [152].

*(3) Reduce TAMs directly*. Tribetidine is an anticancer agent for the treatment of soft tissue sarcomas and platinum-sensitive relapsed ovarian cancer (Table 3) [133, 134]. Tribetidine can activate caspase-8, a crucial component of the exogenous apoptosis pathway, thereby activating the exogenous apoptosis pathway and subsequently inducing TAMs apoptosis [153]. Zoledronic acid (ZA) is an effective nitrogen-containing bisphosphonate (NBP), which not only directly induces the apoptosis of tumor cells but also reduces the *in vivo* amount of TAMs and facilitates the transformation of TAMs into M1 macrophages (Table 3) [154, 155]. LEG-3, a legumain sensitive doxorubicin-based prodrug, selectively ablates TAMs and has been shown to inhibit the growth and metastasis of breast cancer (Table 3) [137, 138]. CD204 and folate receptor *β* (FR*β*) are highly expressed by TAMs, so immunotoxins targeting CD204 and FR*β* can also eliminate TAMs.

#### 2.4.2. Adoptive Cell Therapy

Adoptive cell therapy (ACT) is an antitumor therapy in which autologous immune cells are activated in vitro by interleukin-2 (IL-2) as well as other cytokines, amplified to a certain number, and then injected back into the body of cancer patients where they can kill tumor cells in vivo [156, 157]. ACT using autologous tumor-infiltrating lymphocytes (TILs) is currently the most effective treatment for patients with metastatic melanoma, contributing to the tumor regression in about 50% of patients [158]. TILs are composed of a variety of CD3+ CD4+ and CD3+ CD8+ T-cells, B cells, and NK cells, among which CD8+ T-cells are characterized by the anticancer cytotoxic effect [159]. TILs are currently the most commonly used autoimmune cells of ACT around the world. T-cell receptor-gene engineered T-cells (TCR-T) and chimeric antigen receptor T-cell immunotherapy (CAR-T) are mainly used to improve the function of TIL [156]. Compared with radiotherapy or chemotherapy, ACT has a longer duration of efficacy and works safer. However, TIL contains several components that inhibit the immune response, for instance, Treg cells [160, 161]. Therefore, it is necessary to consider how to remove components such as Treg cells in TIL that can suppress the immune response before immunotherapy is applied. On the other hand, some studies have found that the amount of TIL is only 0.01% of the original amount after it is transferred back into the patients after the blood circulation, which account for the limited therapy efficacy [162]. In the future, the combination of ACT with traditional treatment methods such as surgery, radiotherapy, and chemotherapy will become the trend of tumor therapy. At the same time, it is also necessary to research and discover more effective drugs for combined application.

#### 2.4.3. Targeted Drug Therapy

*(1) Immune checkpoint inhibitors*. Immune checkpoint therapy enhances antitumor immune response through regulating the molecules of signaling pathways in T-cells rather than tumor cells. Till now, three immune checkpoint inhibitors have been approved by the FDA for the treatment of melanoma, including ipilimumab against cytotoxic T lymphocyte-associated antigen 4 (CTLA-4), as well as pembrolizumab and nivolumab against programmed cell death protein 1 (PD-1) [158].

CTLA-4 is a transmembrane receptor predominantly expressed on cytotoxic T-cells which can bind to two ligands CD80 (B7-1) and CD86 (B7-2) on the surface of antigen-presenting cells and suppress the production of IL-2 and the activation of downstream kinase cascade signaling pathways involved in immune response stimulation, thereby inhibiting the activation and anticancer functions of T-cells [163, 164]. Ipilimumab is a monoclonal antibody against CTLA-4 that can competitively bind to CTLA-4 to block the interaction between CTLA-4 and its ligands, thus blocking the inhibitory signals generated in cytotoxic T-cells and enhancing their anticancer activities (Table 3) [139]. The efficacy of ipilimumab in patients with melanoma has been confirmed by multiple clinical trials [140, 141], and the application of ipilimumab in metastatic renal cancer [142], glioblastoma [139], and many other cancer types is also under investigation. However, patients treated with ipilimumab have been reported to experience a number of adverse reactions that mainly manifest as gastrointestinal reactions, including colitis and hepatitis [165].

PD-1 is also a surface receptor expressed on a variety of immune cells such as T cells, B cells, DCs, and NK cells, and it can bind two ligands programmed death-1 ligand (PD-L1) and programmed death-2 ligand (PD-L2) and cause the dephosphorylation of several key molecules in the TCR signaling pathway, thus inhibiting the proliferation and activation of T-cells [49]. Cancer cells have the capability of impairing the cytotoxicity of effector T-cells by activating the PD-1/PD-L1 signaling pathway, which is one of the essential approaches implicated in the immune escape of cancer cells [166]. Therefore, antibodies against PD-1 can block the binding of PD-1 to its ligands, promote the proliferation and activation of T cells, and as a consequence exert an antitumor effect. At present, the antibodies against PD-1 approved for clinical use mainly include nivolumab and pembrolizumab (Table 3). Various clinical trials have demonstrated the efficacy and safety of nivolumab and pembrolizumab in the treatment of melanoma [143, 144]. In addition, nivolumab and pembrolizumab are also used in the treatment of metastatic non-small cell carcinoma and renal cell carcinoma [17].

Compared with traditional chemotherapy, the curative effect of immune checkpoint inhibitors is better and adverse reactions during treatment are less, which have dramatically changed the treatment of malignant tumors. However, more studies are needed to determine the optimal patients for the immune checkpoint inhibitor treatment.

*(2) Multitarget drugs*. Curcumin is a natural compound derived from the curcuma longa, which has been identified to impair multiple signaling pathways and inhibit the proliferation, invasion, metastasis, and angiogenesis of tumor cells (Table 3) [167]. Curcumin is a multitarget drug, which not only regulates the proliferation and the activation of T-cells by inhibiting the expression of IL-2 and NK-*κ*B but also inhibits the growth of tumors by enhancing the activity of CD8+ T-cells [168]. In addition, curcumin can inhibit TGF-*β*, IDO, and some other immunosuppressive factors and increase the recruitment of T-cells, which is conducive to antitumor therapy [169].

The extra domain B (ED-B) of fibronectin can be expressed in specific solid tumor neovascular regions and extracellular matrix but not in normal tissues [170]. ED-B is highly expressed in gliomas [171], and as ED-B is continuously produced during the formation, proliferation, and migration of glioma cells, it is theorized that the higher the grade of glioma, the higher the content of ED-B in tumor neovasculars. Because the physiological function of ED-B is unclear and it is suitable only as a tumor marker, a small fraction of the antibody drugs that have been developed are produced by fusing protein drugs or conjugated with other drug molecules, known as the armed antibody [172]. L-19 [173] is a full human single-chain antibody to ED-B screened by phage display technology, which can be genetically recombined with IL-2, TNF-a, interferon, etc., to form a fusion protein. It can be used for head and neck cancer, diffuse large B cell lymphoma, non-small cell carcinoma, and so on.

In the immune therapy of tumors, it is of significance to find practical biomarkers to guide the choice of effective drugs in order to ensure that patients can achieve the maximal benefit from clinical treatment.

## 3. Discussion

With the extensive studies on TME, antitumor therapy targeting TME has emerged as an exciting prospect. However, there are still some difficulties and challenges in the clinical application of antitumor therapy targeting the TME. First of all, many drugs are only clinically applied to target one specific type of cancer. Moreover, numerous preclinical and clinical trials are exploring their applications in many other cancer types, which hopefully would expand the use of these anticancer therapies. It is also necessary to further identify the pharmacological mechanisms of these agents, in order to improve the application of the drugs in the treatment of multiple malignancies. Although patients indeed receive enormous benefit from the anticancer therapy, the adverse effects of these agents and the development of drug resistance remain to be the obstacles in cancer treatment. A mounting number of studies are investigating the methods to mitigate the side effects, and novel therapeutic chemicals have been developed to overcome the resistance against current agents. Some treatment methods can significantly improve the antitumor effects through a single immunosuppression targets; however, the TME exits as a dynamic regulatory network which is composed of diverse immunosuppression signals generated by many cell types and molecules. Once an individual immunosuppressive signal is blocked or deleted, “smart” tumor cells are capable of evolving other immunosuppressive mechanisms to attenuate the curative effect of therapeutics. Therefore, combination therapy is considered as the trend of future antitumor therapy. Furthermore, animal models of TME are relatively difficult to establish compared with the animal models used in other fields, for example, drug safety evaluation. Therefore, it is also an important direction for future research to establish the animal models that are highly similar to TME *in vivo*, especially one that can simulate the function of various components of TME.

## Figures and Tables

**Table 1:** Pharmacological agents targeting tumor angiogenesis.

Therapeutic agent	Therapeutic agent description	Cancer type	References
Bevacizumab	VEGF antibody	Non-small cell lung carcinoma	[10–16]
		Renal cancer	
		Metastatic colorectal cancer	
		Metastatic breast cancer	

Ramucirumab	VEGFR antibody	Gastric cancer	[17, 18]
		Non-small cell lung carcinoma metastatic colorectal cancer	

Aflibercept	VEGF blocking agent	Advanced solid tumors	[19, 20]
		Colorectal cancer	
		Non-small cell lung cancer	

Pazopanib	Multitarget tyrosine kinase receptor inhibitor	Metastatic renal cell carcinoma advanced soft tissue sarcomas	[21, 22]

Sunitinib	Multitarget tyrosine kinase receptor inhibitor	Advanced gastrointestinal stromal tumors	[23, 24]

Sorafenib	Multitarget tyrosine kinase receptor inhibitor	Inoperable hepatocellular carcinoma	[25, 26]

Regorafenib	Multikinase inhibitor	Metastatic colorectal cancer advanced gastrointestinal	[27, 28]

Everolimus	mTOR inhibitor	Metastatic breast cancer	[29–31]
		Gastric cancer	

Topotecan	Topoisomerase I inhibitor	Small cell lung cancer, ovarian cancer, and cervical cancer	[32, 33]

**Table 2:** Pharmacological agents targeting ECM.

Therapeutic agent	Therapeutic agent description	Cancer type	References
Losartan, valsartan, and their analogs	Anangiotensin receptor blockers	Gastroesophageal cancer	[74–76]
		Pancreatic ductal adenocarcinoma	
		Breast cancer	

Col-3	MMPs inhibitors	—	[22, 23]

Metformin	Remodel ECM	Colorectal cancer	[77, 78]
		Cervical cancer	

**Table 3:** Pharmacological agents targeting the immune system.

Therapeutic agent	Therapeutic agent description	Cancer type	References
Bindarit	CCL2 inhibitors	Bone cancer	[128, 129]
		Prostate cancer	
		Breast cancer	

Celecoxib	COX-2 inhibitors	Colon cancer	[130, 131]
		Breast cancer	
		Prostate cancer	
		Head and neck cancer	

Etodolac	COX-2 inhibitors	Breast cancer	[132]

Tribetidine	Caspase-8 activators	Soft tissue sarcomas	[133, 134]
		Platinum-sensitive relapsed ovarian cancer	

Zoledronic acid	Nitrogen-containing bisphosphonates	Cervical cancer	[135, 136]
		Prostate cancer	

LEG-3	Legumain sensitive doxorubicin-based prodrug	Breast cancer	[137, 138]

Ipilimumab	CTLA-4 antibody	Melanoma	[139–142]
		Metastatic renal cancer	
		Glioblastoma	

Nivolumab/pembrolizumab	PD-1 antibody	Melanoma	[17, 143, 144]
		Metastatic non-small cell carcinoma renal cell carcinoma	

Curcumin	Multitarget drugs	Lung cancer	[131, 145]
		Non-small cell lung carcinoma	

## Additional Points

*Text*. As a critical hallmark of cancer, the tumor microenvironment (TME) has evolved as an important anticancer therapeutic target. Many great efforts have been made to elucidate the roles of TME in tumorigenesis and cancer progression. Although the complexity of TME remains to be a conundrum of the effective targeted therapy, scientists have succeeded in developing a variety of pharmacological interventions to impede the TME functions implicated in tumor malignancies.
